# Supplementary material for: Leveraging spatial-angular redundancy for self-supervised denoising of 3D fluorescence imaging without temporal dependency
Source: Nat Commun. 2025 Nov 24;16:11608. doi: 10.1038/s41467-025-66654-3 (PMC12749375; doi:10.1038/s41467-025-66654-3)
Supplement: Supplementary file 1 — Supplementary Information [file 41467_2025_66654_MOESM1_ESM.pdf]

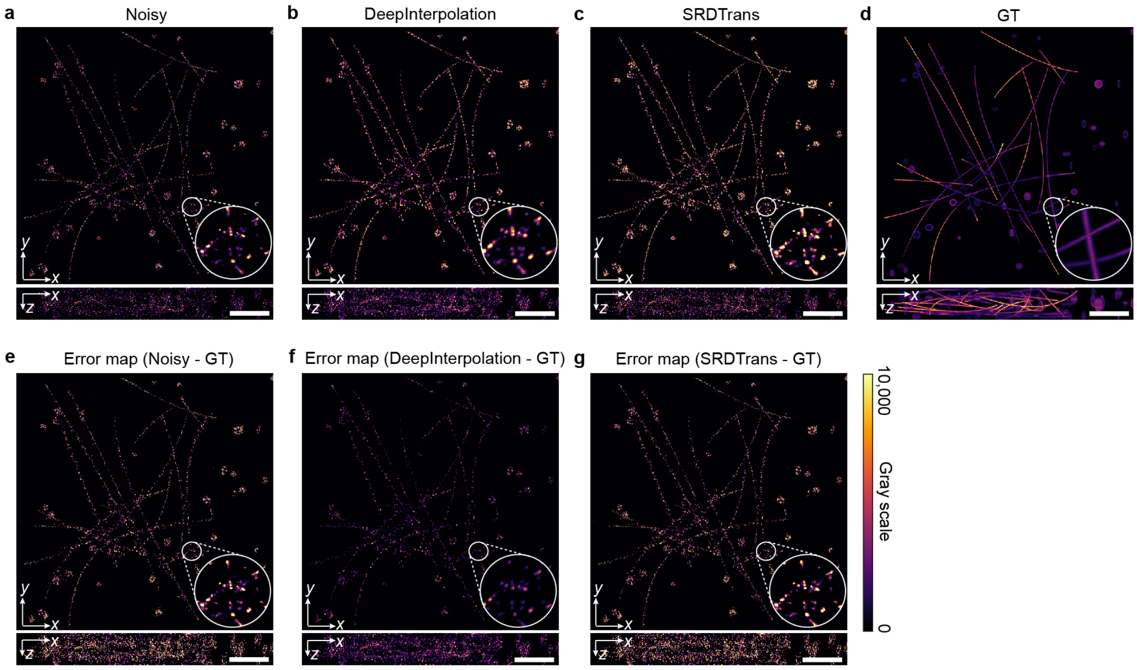

Supplementary Fig. 1 | Denoising performance on volumetric data after 3D reconstruction. a, Simulated tubulins and bubbles with severe noise. **b-c**, Denoising results using DeepInterpolation and SRDTrans. **d**, The corresponding ground truth. **e-g**, Error maps of different methods compare to ground truth, highlighting substantial noise residue. Scale bars, 10 µm.

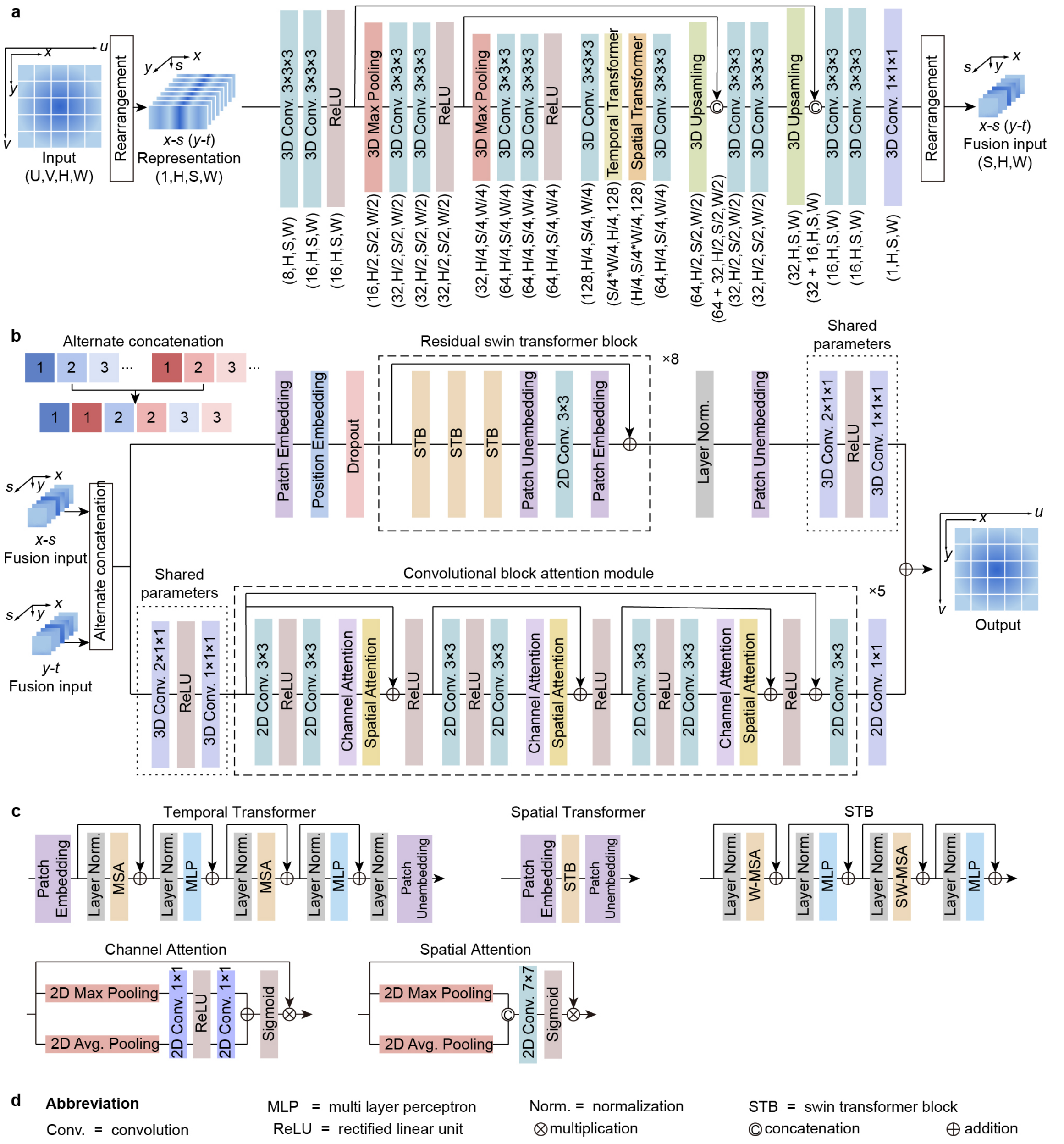

Supplementary Fig. 2 | Detailed network architecture of LF-denoising. a, Detailed structure of the EPI denoising network for single *x-s* or *y-t* path, featuring a series of 3D convolution and spatial-temporal attention for self-supervised learning. **b**, The detailed structure of attention-based fusion module. Two features are fused through alternate concatenation, followed by five convolutional block attention modules and eight residual swin transformer blocks, to generate final features in the spatial-angular domain. **c**, Unfolded structures of temporal transformer, spatial transformer, swin transformer, spatial attention and channel attentional modules. **d**, Symbol meanings and text abbreviations.

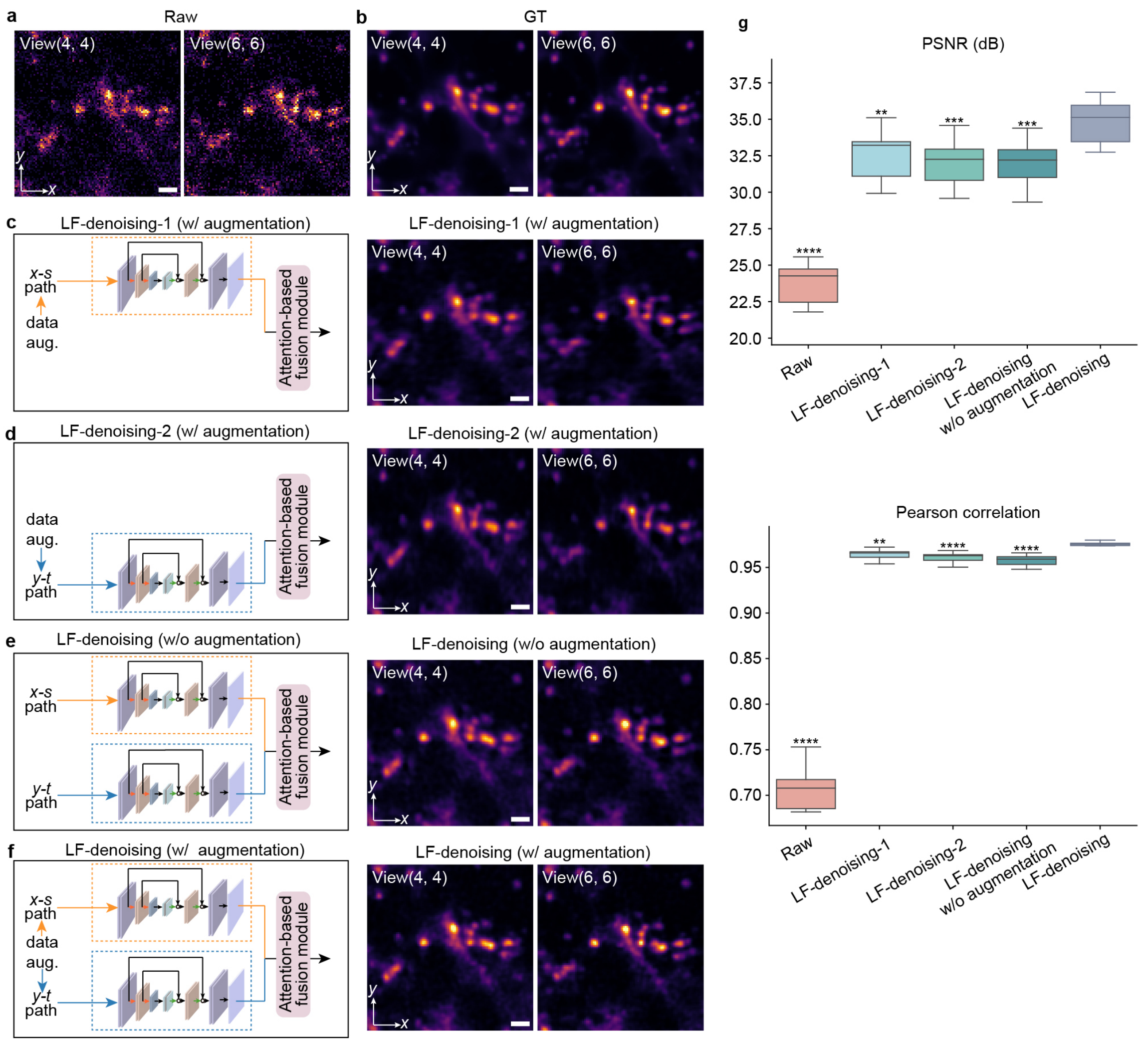

Supplementary Fig. 3 | Ablation study on LF-denoising. a, Noisy angular measurements. **b**, Ground truth without noise. **c**, Simplified schematic of LF-denoising-1, using only *x-s* directional EPI for self-supervision. Angular images after LF-denoising-1 are shown. **d**, Simplified schematic of LF-denoising-2, using only *y-t* directional EPI for self-supervision. Angular images after LF-denoising-2 are shown. **e-f**, Simplified schematic of LF-denoising, using bidirectional EPIs for self-supervision. Data augmentation is used in **f**. Angular images after LF-denoising are shown. **g**, Boxplot showing PSNR and Pearson correlation indices for different methods. The boxplot formats: center line, median; box limits, lower and upper quartiles; whiskers, 1.5-fold interquartile range. All P values were calculated using one-sided independent *t*-test, significance at $P < 0.05$. *$P < 0.05$, **$P < 0.01$, ***$P < 0.001$, ****$P < 0.0001$. $n = 10$ individual samples. P values are 2.47×10^{-7}, 8.43×10^{-3}, 1.89×10^{-3} and 2.41×10^{-3} from left to right for PNSR, and 5.35×10^{-9}, 1.01×10^{-3}, 8.13×10^{-5} and 2.17×10^{-5} from left to right for Pearson correlation. The asterisks indicate the significant levels of comparisons between each method and LF-denoising. Scale bars, 5 μm (**a-f**).

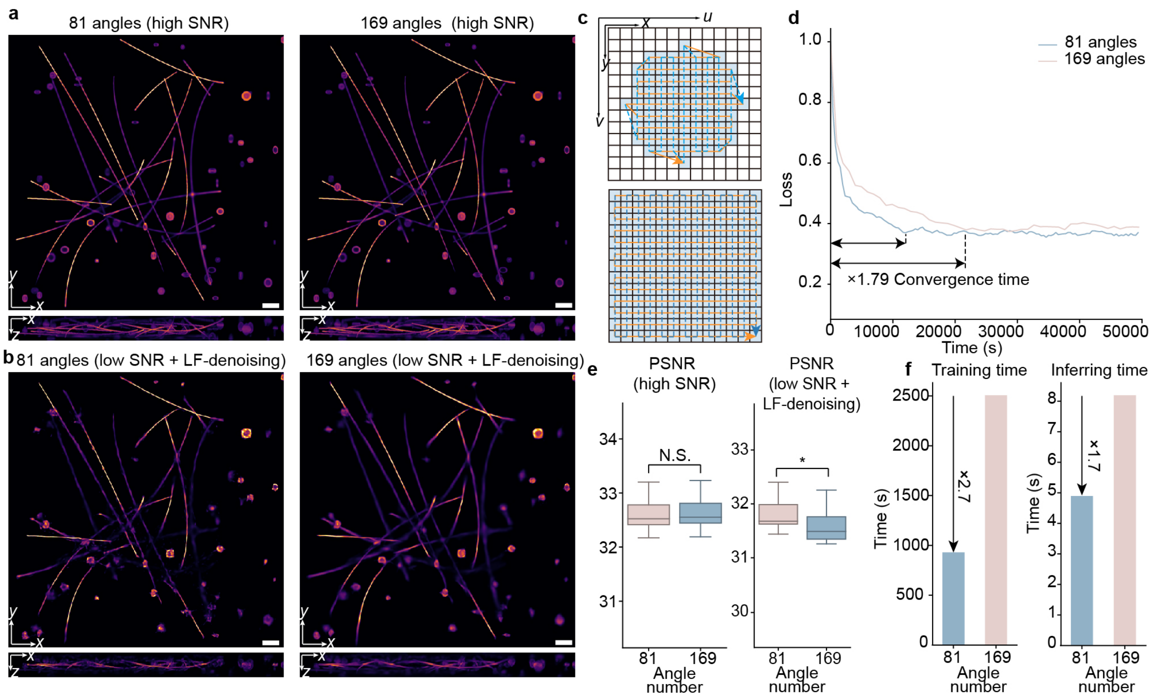

Supplementary Fig. 4 | Angle selection strategy for LF-denoising. a, Reconstruction results for high-SNR input data, using all 81 angles (left) and 169 central angles (right). **b**, Reconstruction results for low-SNR input data after LF-denoising, using 81 angles (left) and 169 angles (right). **c**, Strategy of bidirectional angular traversals to establish EPI data pairs, for 81 angles (top) and 169 angles (bottom). **d**, Convergence curves of LF-denoising with different number of angles used for training. **e**, PSNR metrics for reconstructions of high-SNR input data (left) and low-SNR input data after LF-denoising (right), using 169 angles and 81 angle. All P values were calculated using two-sided independent *t*-test, significance at $P < 0.05$. *$P < 0.05$, **$P < 0.01$, ***$P < 0.001$, ****$P < 0.0001$. $n = 9$ individual samples. P values are 9.88×10^{-1} and 4.76×10^{-2} from left to right. **f**, Comparison of processing time for LF-denoising using different number of angles. Scale bars, 10 μm (**a-b**).

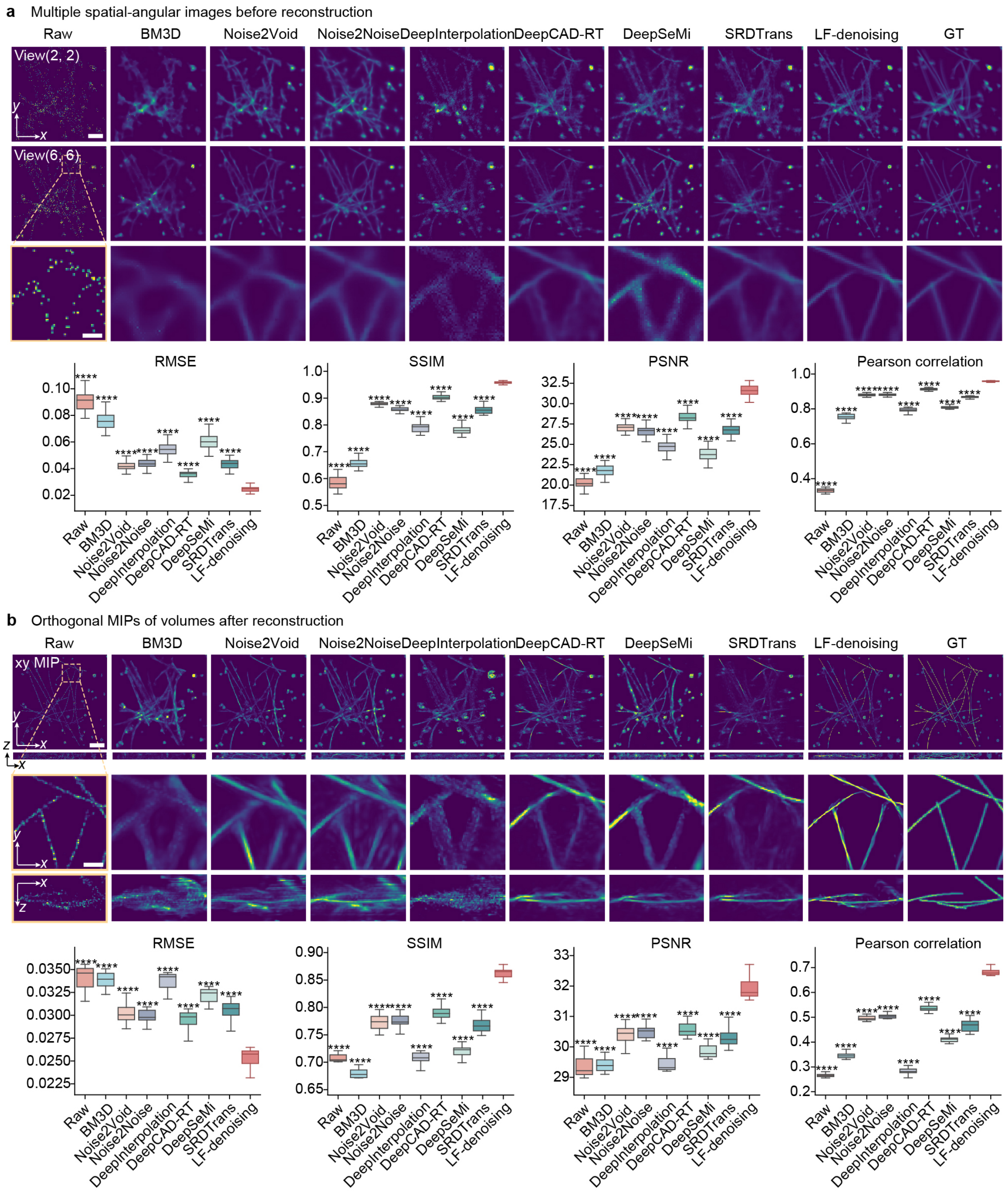

Supplementary Fig. 5 | Benchmark of LF-denoising against other methods before and after reconstruction. a, Example angular views of simulated tubulins and bubbles captured by sLFM with severe noise are shown in the left column. Image bit depth is set to 16, variance of Gaussian noise to 5, and photon number of the maximum intensity to 10. Results processed by BM3D, noise2void, noise2noise, DeepInterpolation, DeepCAD-RT, DeepSeMi, SRDTrans and our LF-denoising are shown in the middle columns. Right column shows the ground truth without noise. Boxplots compares different methods in terms of RMSE, SSIM, PSNR, Pearson correlation. The boxplot formats: center line,

median; box limits, lower and upper quartiles; whiskers, 1.5-fold interquartile range. All P values were calculated using one-sided independent *t*-test, significance at $P < 0.05$. *$P < 0.05$, **$P < 0.01$, ***$P < 0.001$, ****$P < 0.0001$. $n = 15$ individual samples. P values are 1.75×10^{-17}, 8.99×10^{-17}, 5.96×10^{-14}, 1.69×10^{-14}, 6.53×10^{-15}, 7.09×10^{-11}, 1.36×10^{-14}, 1.44×10^{-14}, 4.81×10^{-19}, 7.79×10^{-21}, 5.75×10^{-18}, 3.45×10^{-18}, 9.04×10^{-17}, 1.90×10^{-15}, 1.90×10^{-15}, 5.62×10^{-16}, 1.95×10^{-25}, 6.38×10^{-24}, 2.66×10^{-15}, 3.28×10^{-16}, 1.26×10^{-19}, 9.64×10^{-12}, 6.74×10^{-21}, 4.66×10^{-16}, 1.11×10^{-28}, 1.34×10^{-17}, 8.90×10^{-19}, 3.36×10^{-19}, 3.05×10^{-20}, 1.22×10^{-17}, 2.67×10^{-22} and 7.53×10^{-20} from left to right for metric evaluation. The asterisks indicate the significant levels of comparisons between each method and LF-denoising. **b**, Corresponding results after reconstruction, displayed as orthogonal MIPs. P values are 1.12×10^{-18}, 6.70×10^{-21}, 5.98×10^{-14}, 3.91×10^{-14}, 2.85×10^{-20}, 2.08×10^{-12}, 2.16×10^{-18}, 3.33×10^{-14}, 1.02×10^{-24}, 6.22×10^{-23}, 3.24×10^{-16}, 5.50×10^{-18}, 4.02×10^{-25}, 4.43×10^{-17}, 3.00×10^{-20}, 3.75×10^{-17}, 4.58×10^{-19}, 2.54×10^{-18}, 1.33×10^{-13}, 4.99×10^{-13}, 1.09×10^{-18}, 4.34×10^{-12}, 1.63×10^{-16}, 5.05×10^{-14}, 3.89×10^{-29}, 4.46×10^{-31}, 1.09×10^{-14}, 1.27×10^{-23}, 1.27×10^{-32}, 7.52×10^{-21}, 1.44×10^{-28} and 5.63×10^{-20} from left to right. The asterisks indicate the significant levels of comparisons between each method and LF-denoising. Scale bars, 20 μm (original view) and 5 μm (enlarged view).

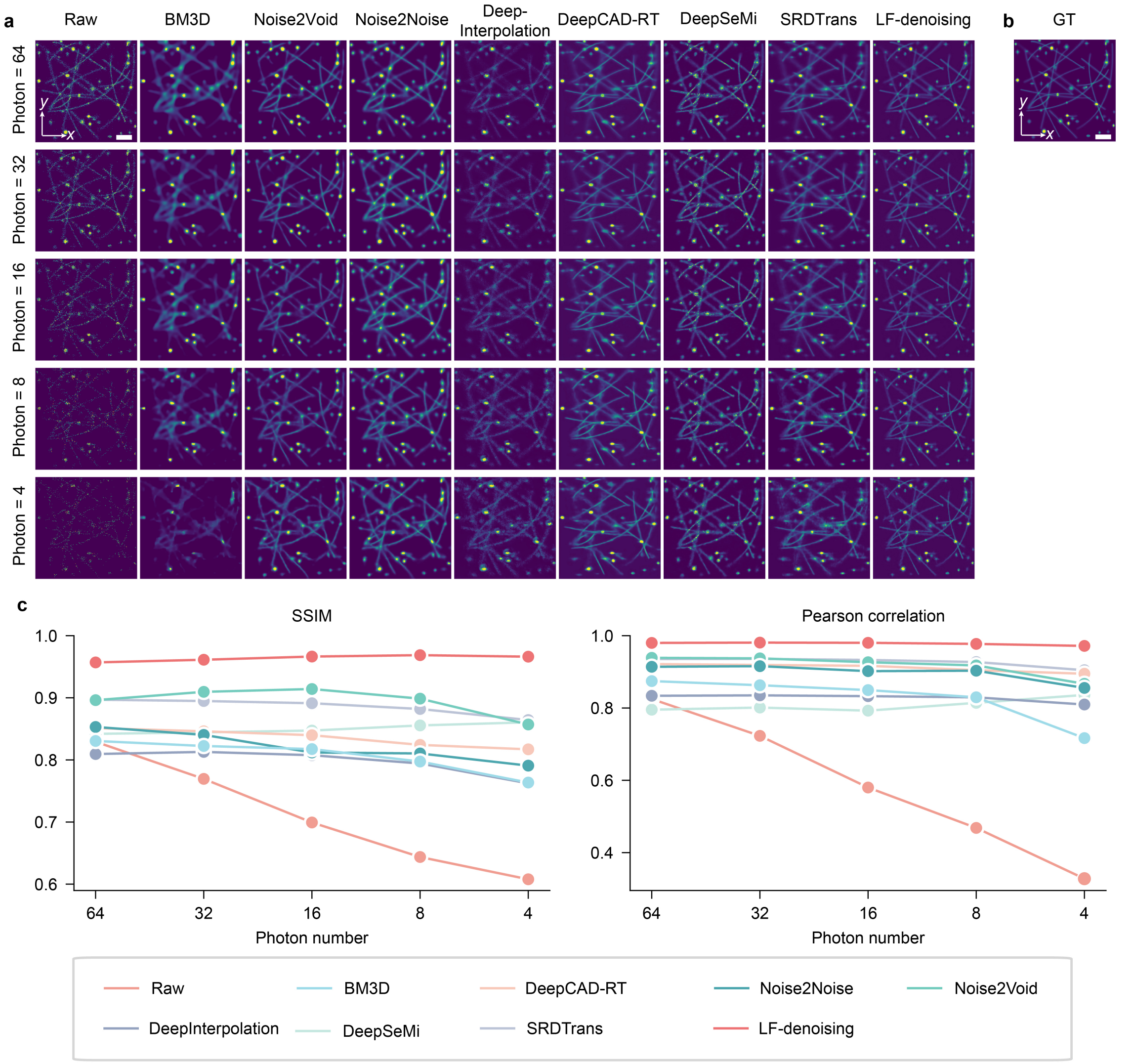

Supplementary Fig. 6 | Benchmark of LF-denoising against other methods for increasing Poisson noise. **a**, Center views of simulated tubulins and bubbles captured by sLFM with increasing Poisson noise are shown in the left column. The noise levels are indicated by maximum detected photon numbers. Results processed by BM3D, noise2void, noise2noise, DeepInterpolation, DeepCAD-RT, DeepSeMi, SRDTrans and our LF-denoising are presented in the following columns. **b**, Ground truth without noise. **c**, Curves of SSIM and Pearson correlation versus varying photon numbers applied for different methods. Scale bars, 20 µm (a, b).

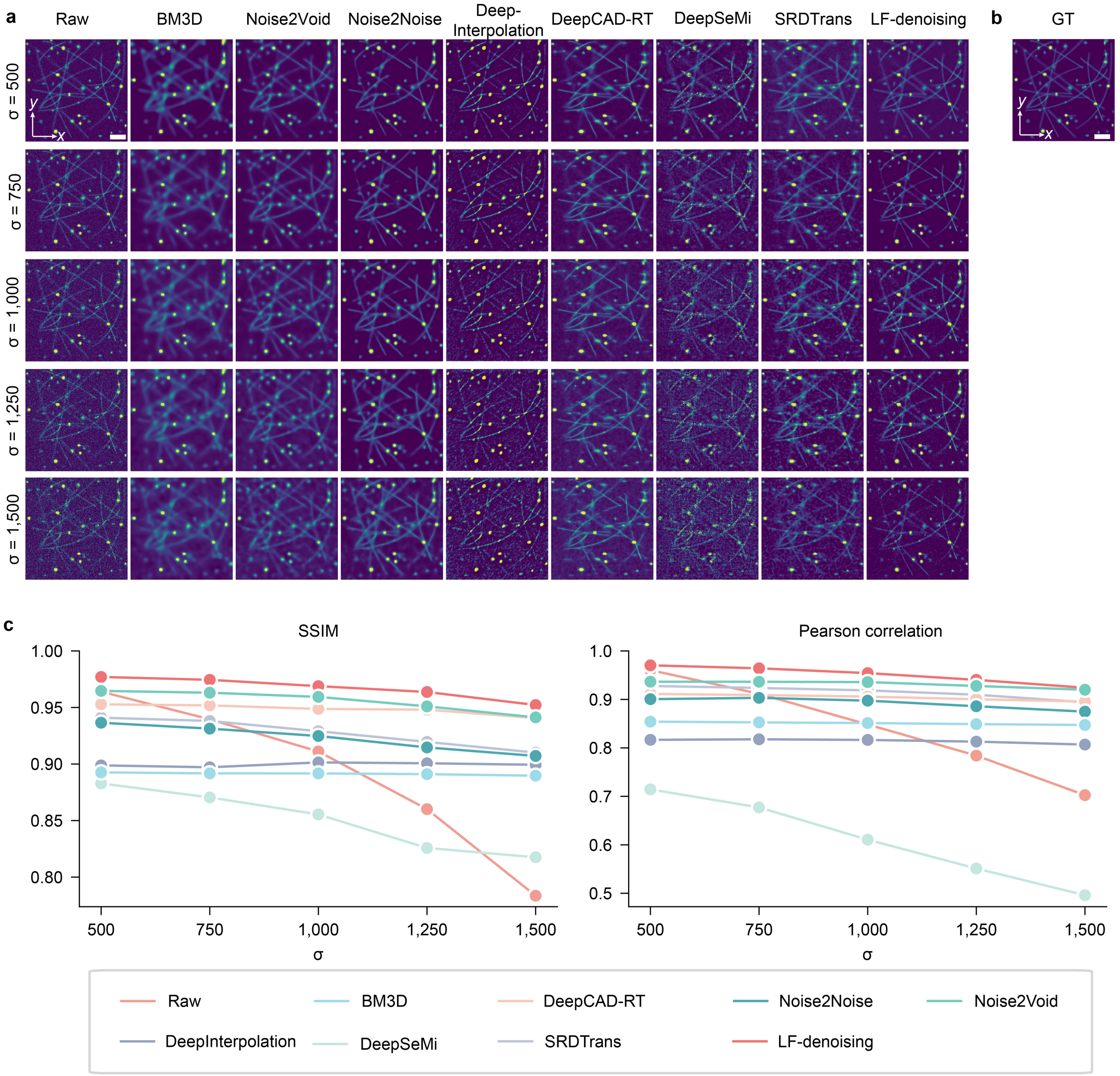

Supplementary Fig. 7 | Benchmark of LF-denoising against other methods for increasing Gaussian noise. a, Center views of simulated tubulins and bubbles captured by sLFM with increasing Poisson noise are shown in the left column. The noise levels are indicated by Gaussian standard deviations σ, with the image bit depth of 16. Results processed by BM3D, noise2void, noise2noise, DeepInterpolation, DeepCAD-RT, DeepSeMi, SRDTrans and our LF-denoising are presented in the following columns. **b**, Ground truth without noise. **c**, Curves of SSIM and Pearson correlation versus varying Gaussian standard deviations applied for different methods. Scale bars, 20 μm (a, b).

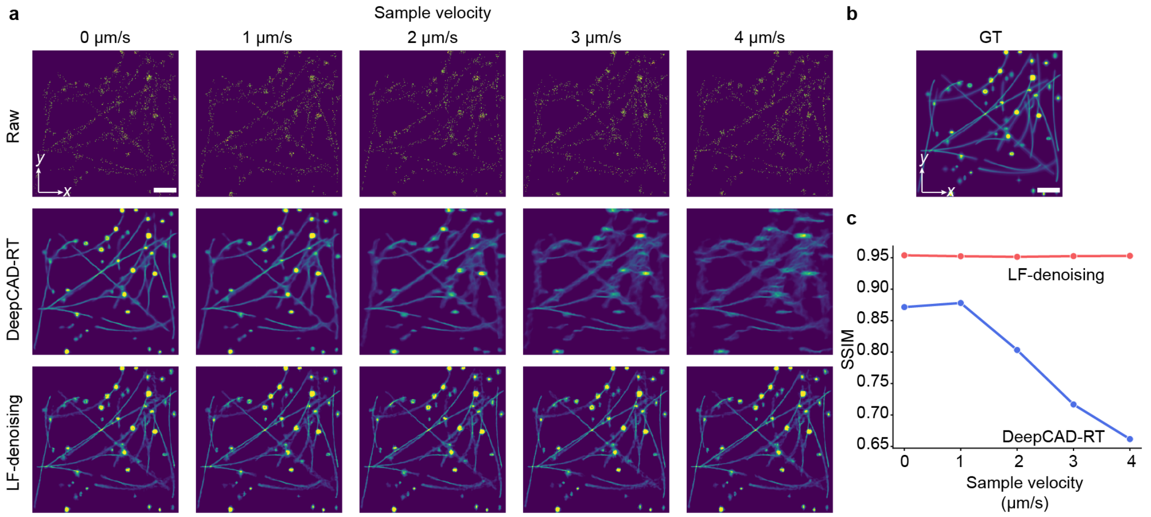

Supplementary Figure 8 | Comparison between DeepCAD-RT and LF-denoising on varied sample velocities. **a**, Center view measurements of simulated tubulins and bubbles captured by sLFM at increasing sample velocities are shown in the first row. The same sample was translated horizontally from left to right. Image bit depth is set to 16, variance of Gaussian noise to 5, and photon number of the maximum intensity to 10, the imaging speed to 42 Hz. Results processed by DeepCAD-RT and LF-denoising are shown in subsequent rows. **b**, Ground truth without noise. **c**, Curves of SSIM versus varying sample velocities applied for DeepCAD-RT and LF-denoising. Scale bars, 20 µm (a, b).

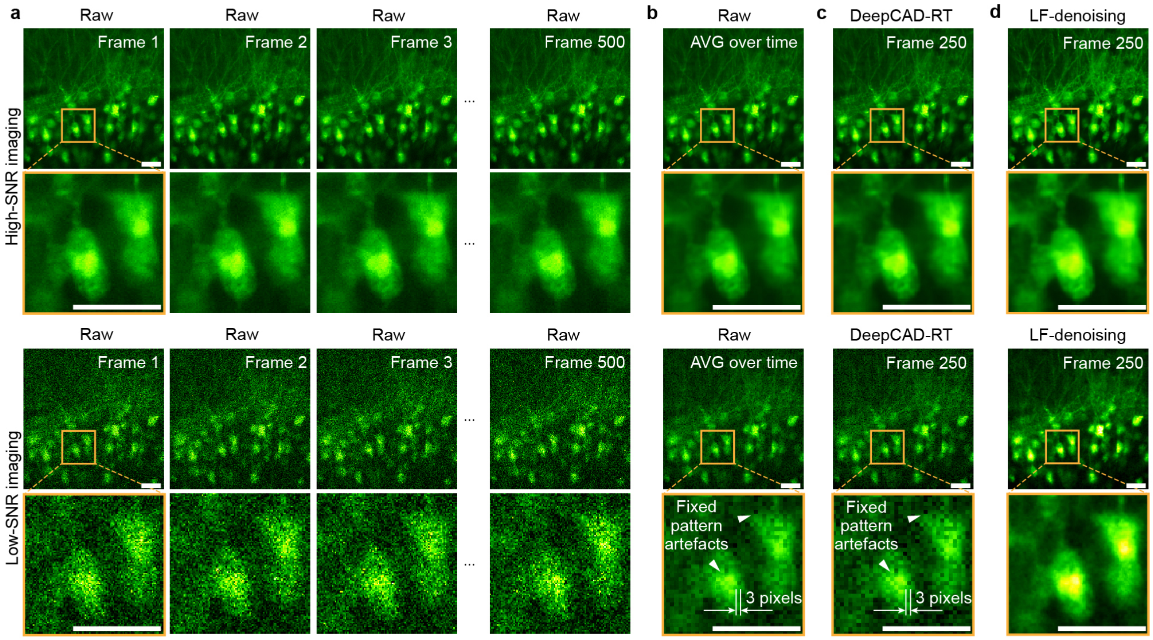

Supplementary Fig. 9 | Temporal dependency causes fixed pattern artefacts in low-SNR sLFM imaging. a-d, Center views of a Thy1-YFP mouse brain slice in high-SNR imaging condition (upper row) and low-SNR imaging condition (lower row) for the raw timelapse recordings in **a**, average recording over time in **b**, DeepCAD-RT enhanced frame in **c** and LF-denoising enhanced frame in **d**. All images were acquired by sLFM with 3×3 scanning mode. After pixel realignment, the 3x3 pixel region in the center views comes from the modulation of the same microlens. The transmittance of different microlenses in the MLA is slightly different due to fabrication errors, which is greatly amplified by averaging operators or DeepCAD-RT, resulting in fixed pattern artefacts with nonuniform intensity in low-SNR imaging. In contrast, LF-denoising shows better performance without such artefacts. Scale bars, 30 μm.

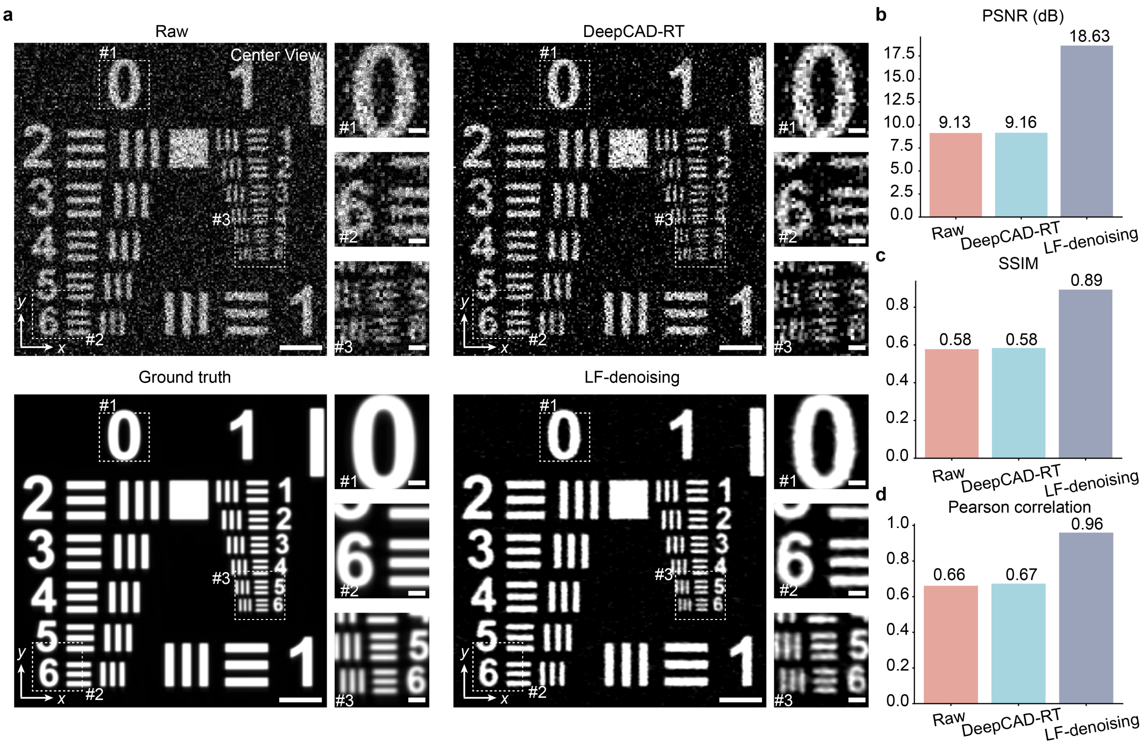

Supplementary Figure 10 | Fix pattern noise removing comparison between DeepCAD-RT and LF-denoising. a, Center view measurements of raw data, ground truth and enhancements by DeepCAD-RT and LF-denoising. **b-d**, Comparison of raw-data and enhancements by DeepCAD-RT and LF-denoising in terms of PSNR (**b**), SSIM (**c**) and Pearson correlation (**d**). Scale bars, 500μm (original view) and 100μm (enlarged view).

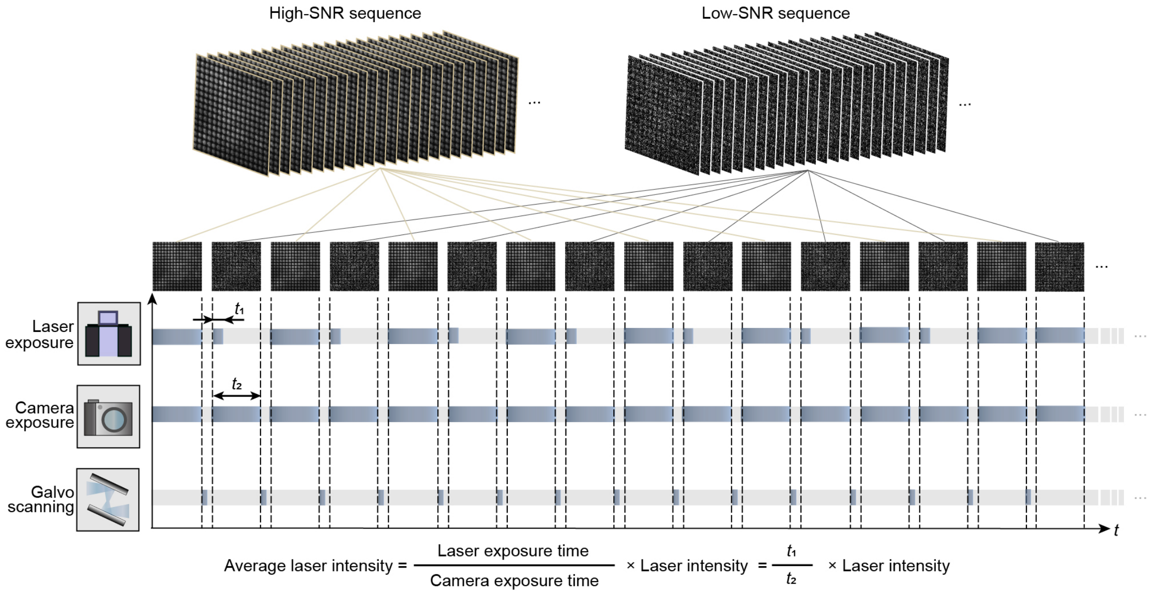

Supplementary Fig. 11 | Hardware control for simultaneously acquisition of synchronized low-SNR and high-SNR sLFM images. During sLFM acquisition, the laser, camera and galvo are synchronously controlled by a customized program. Laser exposure times for odd and even frames are set differently to acquire synchronized low-SNR and high-SNR images. After data acquisition, the raw image sequence is rearranged according to odd-even order to obtain high-SNR and low-SNR sequences. The average laser intensity denotes the mean value during camera exposure.

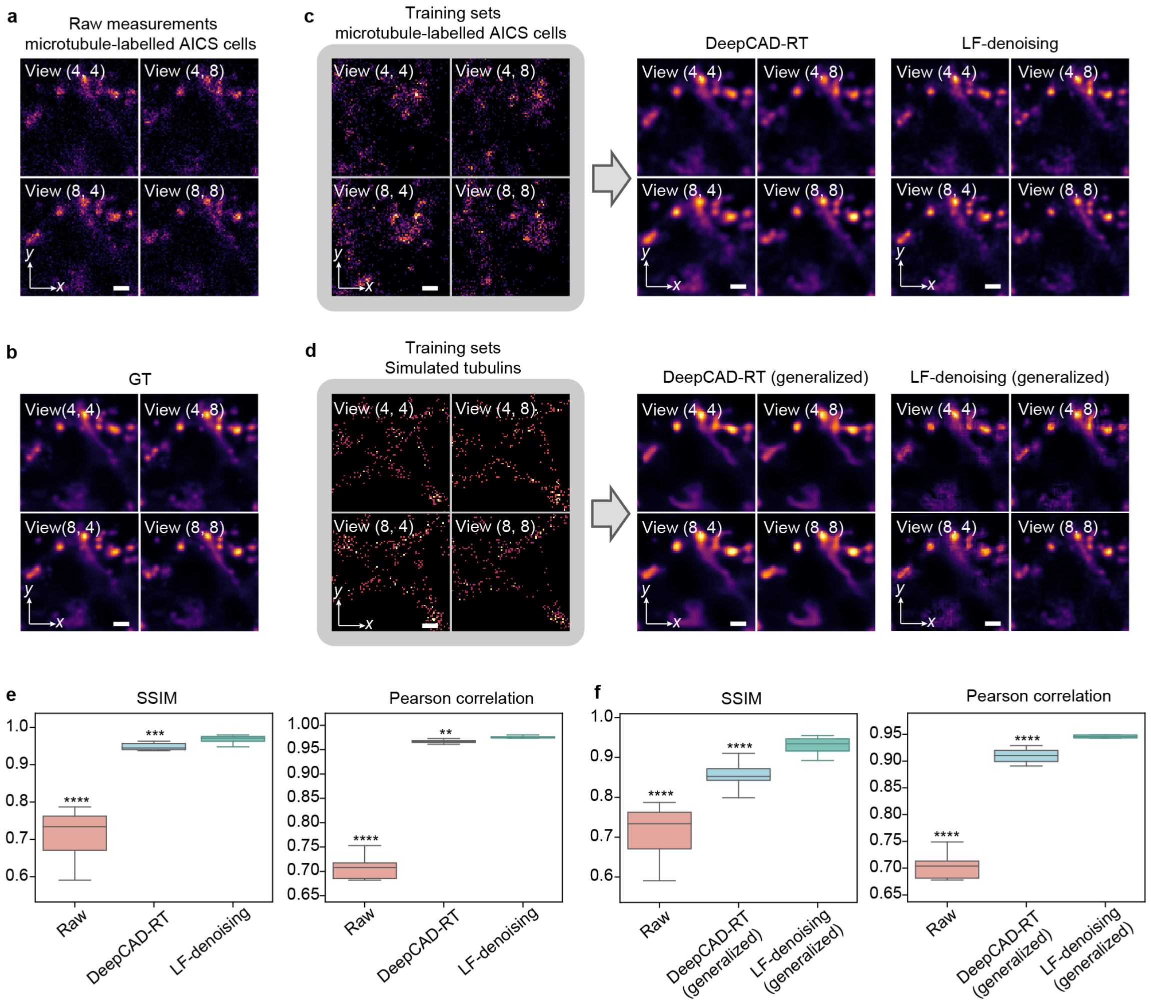

Supplementary Fig. 12 | Generalization test for LF-denoising. **a**, Noisy measurements of microtubule-labelled AICS cells, used as input for generalization test. **b**, Ground truth. **c**, Comparison of models trained on microtubule-labelled AICS cells to enhance the same type of data, comparing DeepCAD-RT and LF-denoising. **d**, Comparison of models trained on synthetic data to denoise microtubule-labelled AICS cells, comparing DeepCAD-RT and LF-denoising. **e-f**, Boxplots comparing different methods in terms of SSIM in **e** and Pearson correlation in **f**. The boxplot formats: center line, median; box limits, lower and upper quartiles; whiskers, 1.5-fold interquartile range. All P values were calculated using one-sided independent *t*-test, significance at $P < 0.05$. *$P < 0.05$, **$P < 0.01$, ***$P < 0.001$, ****$P < 0.0001$. $n = 10$ individual samples. P values are 2.09×10^{-7}, 5.54×10^{-4}, 5.35×10^{-9}, 4.18×10^{-3}, 4.00×10^{-7}, 1.12×10^{-5}, 4.99×10^{-9} and 7.72×10^{-6} from left to right. Scale bars, 5 μm.

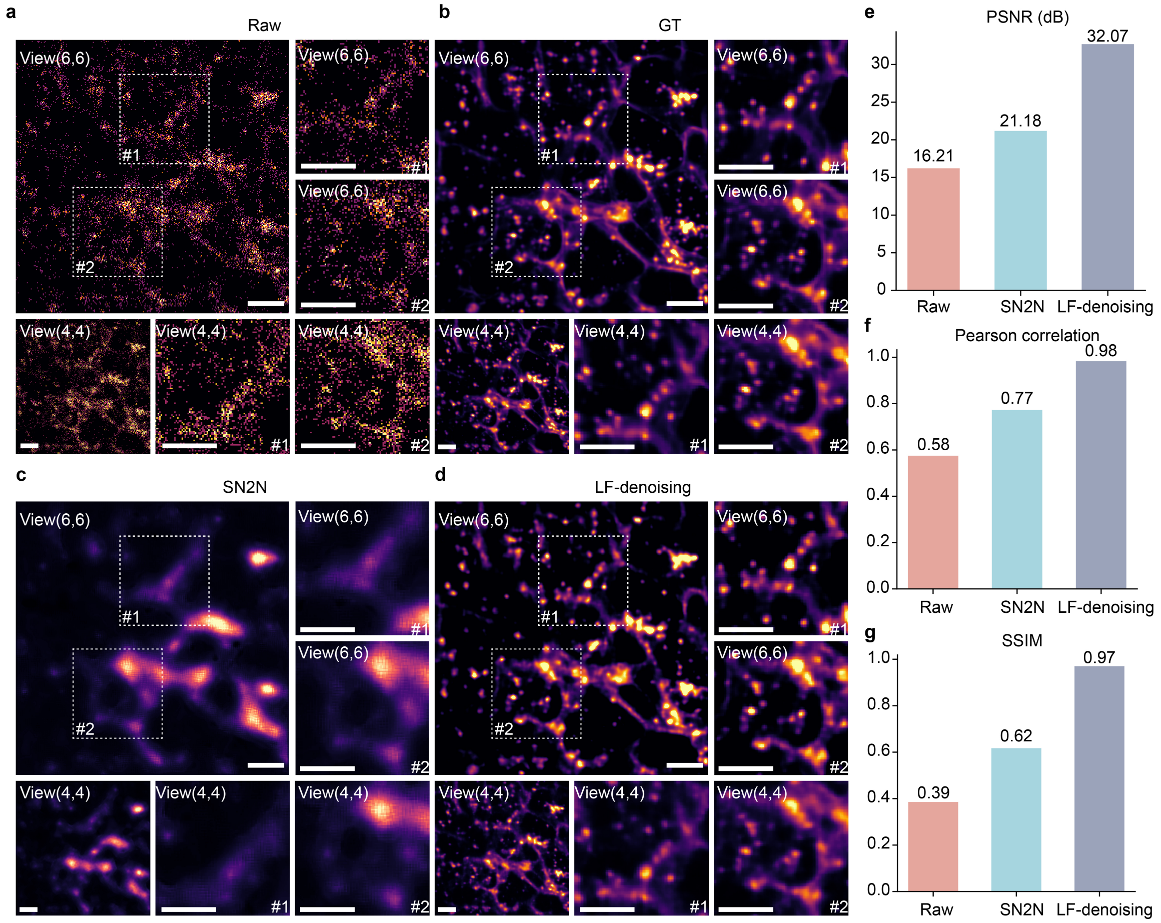

Supplementary Fig. 13 | Comparison between LF-denoising and SN2N. a-d, Angular measurements of raw data in **a**, ground truth in **b**, and enhancements by SN2N in **c** and LF-denoising in **d**. **e-g**, Comparison of raw data and enhancements by SN2N and LF-denoising in terms of PSNR in **e**, Pearson correlation in **f** and SSIM in **g**. Scale bars, 5 µm.

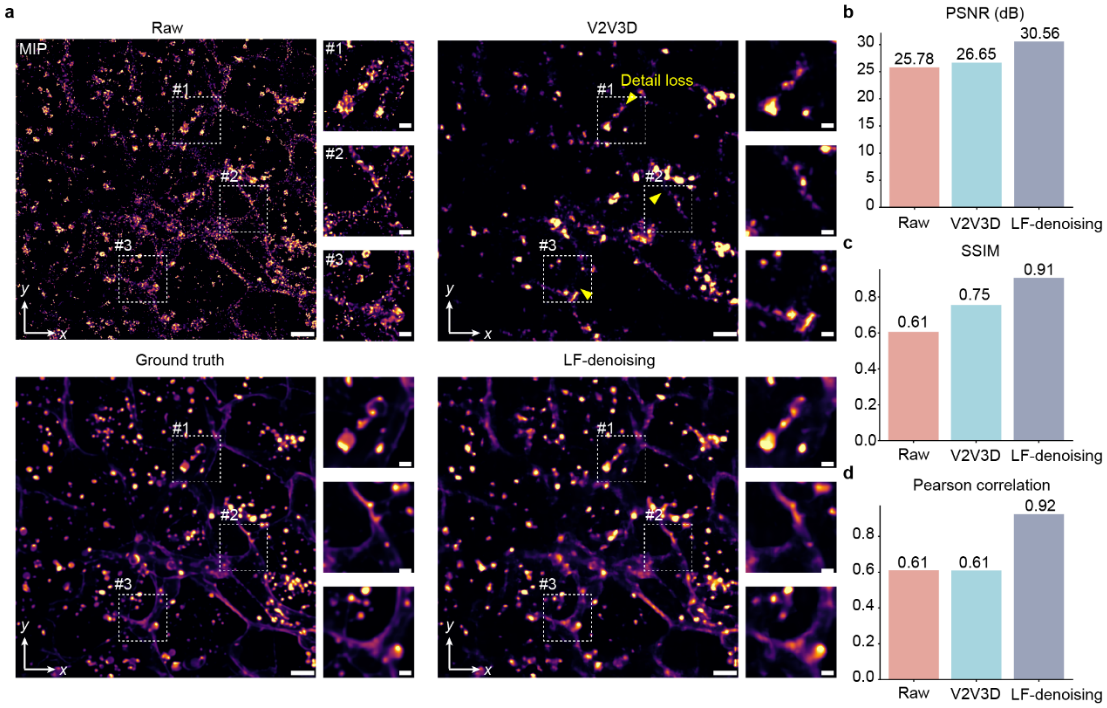

Supplementary Fig. 14 | Comparison between V2V3D and LF-denoising on the data captured by sLFM. a, MIPs of raw data, ground truth and enhancements by V2V3D and LF-denoising after reconstruction. **b-d**, Comparison of raw-data and enhancements by V2V3D and LF-denoising in terms of PSNR (**b**), SSIM (**c**) and Pearson correlation (**d**). Scale bars, 10 µm (original view) and 5 µm (enlarged view).

Supplementary Table 1 | Imaging conditions for all fluorescence experiments

	Sample (imaging T, °C)	Fluorescent label	λ: average power/unit area (mW/mm^2)	Laser exposure time (# time pts)	Volume rate (VPS)	Objective	Setup, angular resolution
3	Zebrafish larva (27 °C)	*Tg(flk:EGFP; gata1:DsRed)*	488: 1.5<br>561: 2.5	10 ms<br>100 pts	50	63×/1.4NA Oil	LFM, inverted, 13 × 13
4	Zebrafish embryo (27 °C)	EGFP	488: 0.01	1 ms<br>36,940 pts	1	63×/1.4NA Oil	sLFM, inverted, 13 × 13
4d (50× SNR)	Zebrafish embryo (27 °C)	EGFP	488: 0.5	50 ms<br>36,940 pts	1	63×/1.4NA Oil	sLFM, inverted, 13 × 13
5a-5f	Living mouse liver (37 °C)	Ly6G<br>WGA	488: 0.07<br>561: 0.08	3 ms<br>455 pts	1/20	63×/1.4NA Oil	sLFM, inverted, 13 × 13
5a-5f (20× SNR)	Living mouse liver (37 °C)	Ly6G<br>WGA	488: 1.4<br>561: 1.6	60 ms<br>455 pts	1/20	63×/1.4NA Oil	sLFM, inverted, 13 × 13
5g-5l	Living mouse brain (37 °C)	GCamp6f	488: 0.01	0.1 ms<br>1,110 pts	30	25×/1.05NA Water	sLFM, upright, 21 × 21
5g-5l (50× SNR)	Living mouse brain (37 °C)	GCamp6f	488: 0.5	5 ms<br>1,110 pts	30	25×/1.05NA Water	sLFM, upright, 21 × 21
6	*Drosophila* brain (27 °C)	jGCaMP7f	920: 5.1	400 ms<br>6,000 pts	2.3	25×/1.05NA Water	2pSAM, upright, 13
S9 (high SNR)	Mouse brain slice (27 °C)	THy1-YFP	488: 1.0	100 ms<br>500 pts	4	63×/1.4NA Oil	sLFM, inverted, 13 × 13
S9 (low SNR)	Mouse brain slice (27 °C)	THy1-YFP	488: 0.01	1 ms<br>500 pts	4	63×/1.4NA Oil	sLFM, inverted, 13 × 13
