# Supplementary material for: Leveraging spatial-angular redundancy for self-supervised denoising of 3D fluorescence imaging without temporal dependency
Source: Nat Commun. 2025 Nov 24;16:11608. doi: 10.1038/s41467-025-66654-3 (PMC12749375; doi:10.1038/s41467-025-66654-3)
Supplement: Supplementary file 2 — Description of Additional Supplementary Files [file 41467_2025_66654_MOESM2_ESM.pdf]

Description of Additional Supplementary Files

Supplementary Movie 1 | Denoising of highly dynamic 3D imaging such as heart beating in zebrafish larvae at 50 VPS

This video presents two directional maximum intensity projections (MIPs) of a beating zebrafish heart, comparing the raw recording with those enhanced by DeepCAD-RT, DeepSeMi, SRDTrans and LF-denoising. The data was captured by LFM with low excitation at 50 volumes per second. Despite the rapid movements of red blood cells and hearts, LF-denoising clearly resolved them with high resolution. Scale bars are included.

Supplementary Movie 2 | Denoising of day-long high-speed observation of 3D membrane dynamics in zebrafish embryos

This video comprises three parts. (**Part Ⅰ**) Photobleaching evaluation. Kymographs and center views of recordings at laser intensities of 500 μW/mm² and 10 μW/mm². High-SNR images photobleached within 1.5 hours, whereas low-intensity observations lasted over 10 hours. (**Part II**) Comparison of raw and enhanced recordings. Data before and after reconstruction using DeepCAD-RT and LF-denoising are shown, highlighting the problem of fixed pattern artifacts on DeepCAD-RT. (**Part III**) 3D formation of migrasomes. The process is clearly presented by LF-denoising. Scale bars are included.

Supplementary Movie 3 | LF-denoising enhances SNR of immune subcellular microenvironments in living mouse livers

This video shows *in-vivo* recordings of neutrophils in mouse liver vessels, comparing low-SNR, high-SNR, and low-SNR enhanced by LF-denoising. Recordings were synchronized in an inverted sLFM system with short and long laser durations. Then, a ROI demonstrating migrasome formation was zoomed in for comparison, showing that LF-denoising effectively reduces fragmented noise and preserves cell morphology. All recordings underwent 3D reconstruction. Scale bars are included.

Supplementary Movie 4 | LF-denoising enhances SNR of 3D neural recordings in mouse cortex *in vivo*

This video presents the raw and LF-denoising enhanced recordings of neural activity in the mouse cortex at 30 VPS. Functional traces of 11 selected neurons are plotted. LF-denoising accurately resolves neural responses, aligning closely with the high-SNR reference. All recordings underwent 3D reconstruction. Scale bars are included.

Supplementary Movie 5 | LF-denoising preserves temporal causality relationships for neural analysis in *Drosophila*

This video compares recordings from a 2pSAM system before and after LF-denoising enhancement, captured from a living *Drosophila* brain under odor stimuli. Functional traces near the moment of odor stimulation are enlarged for comparison. LF-denoising reduces trace fluctuation while maintaining the identical trend of raw data, thus preserving event causal relationships. Scale bars are included.
