# Supplementary material for: Leveraging spatial-angular redundancy for self-supervised denoising of 3D fluorescence imaging without temporal dependency
Source: Nat Commun. 2025 Nov 24;16:11608. doi: 10.1038/s41467-025-66654-3 (PMC12749375; doi:10.1038/s41467-025-66654-3)
Supplement: Supplementary file 8 — Reporting Summary [file 41467_2025_66654_MOESM8_ESM.pdf]

nature portfolio

Corresponding author(s): Jiamin Wu, Qionghai Dai

Last updated by author(s): Oct 28, 2025

Reporting Summary

Nature Portfolio wishes to improve the reproducibility of the work that we publish. This form provides structure for consistency and transparency in reporting. For further information on Nature Portfolio policies, see our Editorial Policies and the Editorial Policy Checklist.

Statistics

For all statistical analyses, confirm that the following items are present in the figure legend, table legend, main text, or Methods section.

n/a	Confirmed	
☐	☒	The exact sample size (n) for each experimental group/condition, given as a discrete number and unit of measurement
☐	☒	A statement on whether measurements were taken from distinct samples or whether the same sample was measured repeatedly
☐	☒	The statistical test(s) used AND whether they are one- or two-sided<br>*Only common tests should be described solely by name; describe more complex techniques in the Methods section.*
☒	☐	A description of all covariates tested
☒	☐	A description of any assumptions or corrections, such as tests of normality and adjustment for multiple comparisons
☐	☒	A full description of the statistical parameters including central tendency (e.g. means) or other basic estimates (e.g. regression coefficient) AND variation (e.g. standard deviation) or associated estimates of uncertainty (e.g. confidence intervals)
☐	☒	For null hypothesis testing, the test statistic (e.g. F, t, r) with confidence intervals, effect sizes, degrees of freedom and P value noted<br>*Give P values as exact values whenever suitable.*
☒	☐	For Bayesian analysis, information on the choice of priors and Markov chain Monte Carlo settings
☒	☐	For hierarchical and complex designs, identification of the appropriate level for tests and full reporting of outcomes
☐	☒	Estimates of effect sizes (e.g. Cohen's d, Pearson's r), indicating how they were calculated

Our web collection on statistics for biologists contains articles on many of the points above.

Software and code

Policy information about availability of computer code

Data collection	LFM and sLFM imaging data were acquired using LABVIEW (2019 version) and our previously published acquisition software, termed sLFdriver (version 2.0, refer to Lu, Z. et al. Nat. Protoc. 2022. https://doi.org/10.1038/s41596-022-00703-9).
Data analysis	All data processing and analyses were performed with customized MATLAB (MathWorks, MATLAB 2019a) scripts and Python (3.7 version) scripts. The 3D volumes of zebrafish larvae were rendered using Imaris (Imaris 9.0.1 software). The 3D rendering of the volumes in supplementary videos was carried out by Voltex modules in Amira (Thermo Fisher Scientific, Amira 2019). The 3D tracking of blood cells in the vessels of the zebrafish larvae were carried out using Imaris.

For manuscripts utilizing custom algorithms or software that are central to the research but not yet described in published literature, software must be made available to editors and reviewers. We strongly encourage code deposition in a community repository (e.g. GitHub). See the Nature Portfolio guidelines for submitting code & software for further information.

Data

Policy information about availability of data

All manuscripts must include a data availability statement. This statement should provide the following information, where applicable:

- Accession codes, unique identifiers, or web links for publicly available datasets
- A description of any restrictions on data availability
- For clinical datasets or third party data, please ensure that the statement adheres to our policy

All relevant data for LF-denoising has been made publicly available on Zenodo (https://doi.org/10.5281/zenodo.11274187).

All relevant codes of LF-denoising are available on GitHub (https://github.com/LF-denoising/LF-denoising) or Zenodo (https://doi.org/10.5281/zenodo.16938935).

Research involving human participants, their data, or biological material

Policy information about studies with human participants or human data. See also policy information about sex, gender (identity/presentation), and sexual orientation and race, ethnicity and racism.

Reporting on sex and gender	No human research participants in this study.
Reporting on race, ethnicity, or other socially relevant groupings	No human research participants in this study.
Population characteristics	No human research participants in this study.
Recruitment	No human research participants in this study.
Ethics oversight	No human research participants in this study.

Note that full information on the approval of the study protocol must also be provided in the manuscript.

Field-specific reporting

Please select the one below that is the best fit for your research. If you are not sure, read the appropriate sections before making your selection.

[x] Life sciences [ ] Behavioural & social sciences [ ] Ecological, evolutionary & environmental sciences

For a reference copy of the document with all sections, see nature.com/documents/nr-reporting-summary-flat.pdf

Life sciences study design

All studies must disclose on these points even when the disclosure is negative.

Sample size	The sample size is labeled in the figure legends. For SSIM demonstrated in Fig. 1, n=30 regions from 12 simulated neuron data were used. For statistical metrics in Fig.2, n=30 for each method. For Pearson correlation comparison in Fig. 5, n=61 fluorescence traces were used. For analysis in Fig. 6, n=20 identified peaks of functional traces were used for each method. For PSNR and SSIM metrics in Supplementary Fig. 3, n=10 individual samples were used. For PSNR in Supplementary Fig. 4, n=9 samples were used for both high-SNR and low-SNR input. For Supplementary Fig. 5, n=15 simulated tubulins and bubbles were used. For Supplementary Fig. 12, n=10 samples were used. The sample sizes were chosen to be sufficiently large following typical practice in related computational imaging studies, ensuring that conclusions remain consistent across regenerated simulation or additional extracted traces.
Data exclusions	No data were excluded for the analysis.
Replication	Biological data shown in Figs. 3-6, Supplementary Fig. 9 and Supplementary Videos 1-5 are representative of n=6 experiments. Biological data shown in Fig. 2 and Supplementary Figs. 3, 12-14 are representative of n=10 experiments. Simulated data shown in Fig. 1 and Supplementary Figs. 1, 4-8, 10 is representative of n=12 experiments.
Randomization	Randomization was not relevant to this study, since no experimental group was formed.
Blinding	Blinding was not relevant to this study, since no group allocation was performed.

Reporting for specific materials, systems and methods

We require information from authors about some types of materials, experimental systems and methods used in many studies. Here, indicate whether each material, system or method listed is relevant to your study. If you are not sure if a list item applies to your research, read the appropriate section before selecting a response.

Materials & experimental systems

n/a	Involved in the study	
[ ]	[x]	Antibodies
[x]	[ ]	Eukaryotic cell lines
[x]	[ ]	Palaeontology and archaeology
[ ]	[x]	Animals and other organisms
[x]	[ ]	Clinical data
[x]	[ ]	Dual use research of concern
[x]	[ ]	Plants

Methods

n/a	Involved in the study	
[x]	[ ]	ChIP-seq
[x]	[ ]	Flow cytometry
[x]	[ ]	MRI-based neuroimaging

Antibodies

Antibodies used	Alexa Fluor 647 Ly6G antibody (Cat# 127610, Lot# B420110, Clone 1A8, 0.5 mg/ml, Biolegend)
Validation	Alexa Fluor 647 Ly6G antibody was validated from the website(https://www.biolegend.com/nl-nl/products/alexa-fluor-647-anti-mouse-ly-6g-antibody-4780), and purchased to perform mouse experiments.

Animals and other research organisms

Policy information about studies involving animals; ARRIVE guidelines recommended for reporting animal research, and Sex and Gender in Research

Laboratory animals	Thy1-YFP-H transgenic mice (Jackson stock No. 003782), Tg(flk:EGFP; gata1:DsRed) transgenic zebrafish larvae at 4 days postfertilization, wild-type (WT) C57BL/6J mice aged 6-8 weeks. Drosophila was of the genotype: w; UAS-rGRAB_ACh-0.5/+; nSyb-Gal4, UAS-jGCaMP7f, combined by w; UAS-rGRAB_ACh-0.5/cyo; +/(TM2/TM6B), nSyb-Gal4 and UAS-jGCaMP7f.
Wild animals	Not involved in this study
Reporting on sex	The mice used in this study are male. The biological sex of zebrafish used in the study is unknown. The Drosophila used in this study are female.
Field-collected samples	Not involved in this study
Ethics oversight	Animal protocol procedures were reviewed and approved by the Institutional Animal Care and Use Committee office of Tsinghua University.

Note that full information on the approval of the study protocol must also be provided in the manuscript.

Plants

Seed stocks	Not involved in this study
Novel plant genotypes	Not involved in this study
Authentication	Not involved in this study
